# Salinity Gradient Modulates the Effects of *Tamarix chinensis* Litter Decomposition on Soil Microbial Community Structure in the Yellow River Delta

**DOI:** 10.3390/plants15192997

**Published:** 2026-09-30

**Authors:** Ying Fang, Yue Lu, Hailin Ma, Xingjian Dun, Chuanqiang Yang, Haidong Xu, Jiangbao Xia

**Affiliations:** 1 Shandong Key Laboratory of Eco-Environmental Science for the Yellow River Delta, Shandong University of Aeronautics, Binzhou 256603, China; fangying6582992@163.com (Y.F.); yue02152023@163.com (Y.L.); donghai@sdua.edu.cn (H.X.); 2 Shandong Academy of Forestry, Jinan 250014, China; mahlin@163.com (H.M.); dxingjian@163.com (X.D.); 3 Shandong Territorial Spatial Planning Institute, Jinan 250013, China; ycq28@163.com

**Keywords:** litter decomposition, salinity gradient, soil microbial community, soil improvement, *Tamarix chinensis*, Yellow River Delta

## Abstract

This study aimed to investigate the influence of *Tamarix chinensis* litter decomposition on soil physicochemical properties and soil microbial community structure under different saline conditions. The experimental design included four salinity treatments: control (CK), slight (SS, 0.4%), moderate (SM, 0.8%), and high (SH, 1.2%). Litter from three decomposition degrees (undecomposed, semidecomposed, and already decomposed) was examined. The litter decomposition rate, 0–10 cm soil physicochemical properties, and soil microbial community structure were measured. The results revealed that as soil salinity increased, a rise was observed in both litter decomposition rate and soil bulk density, whereas soil saturated water content exhibited a declining trend. The soil available nutrients tended to increase overall and were greater under the SM and SH conditions. Compared with CK condition, under SM and SH conditions, the litter decomposition rate increased by 5.05% and 21.01%, respectively, while the soil saturated water content decreased by 6.02% and 5.34%, respectively. The soil NO_3_^−^-N content was 17.54 and 16.45 times that of CK, respectively, whereas the AK content increased by 28.02% and 29.91%, respectively. The soil microbial diversity decreased significantly. Compared with CK, under SM and SH conditions, the bacterial Chao1 index decreased by 14.42% and 17.37%, respectively, and the bacterial Shannon index decreased by 4.46% and 3.82%, respectively. The fungal Chao1 index decreased by 62.52% and 59.99%, respectively, and the fungal Shannon index decreased by 9.57% and 12.79%, respectively. The microbial community structure was similar under the SM and SH conditions, and the relative abundance and number of genera of the dominant phylum were greater. Soil salt content and electrical conductivity (EC) were significantly (*p* < 0.05) or extremely significantly (*p* < 0.01) positively correlated with soil available nutrient content. Soil salinity directly or indirectly affected soil physicochemical properties by regulating the litter decomposition rate, subsequently affecting the soil microbial community structure. The *T. chinensis* litter decomposition enhanced soil available nutrients in moderate and high coastal saline-alkaline soil and the screening ability of the salt-tolerant soil microbial community. These findings offer a scientific reference for the management of low-productivity *T. chinensis* forests and the ecological restoration of saline–alkaline soil in the Yellow River Delta.

## 1. Introduction

Soil salinization is a common resource and ecological environmental problem faced by all countries worldwide. The Yellow River Delta, situated in the warm temperate zone of China, is recognized as one of the most intact, youngest, and extensive coastal wetland ecosystems in the region [1]. The problem of soil salinization caused by seawater intrusion, shallow groundwater levels, high salinity, and high evaporation ratios is serious. At present, the methods to improve saline–alkaline soil in the Yellow River Delta have mainly consisted of the addition of modifiers to saline–alkaline soil and the application of microbial fertilizers [2]. Vegetation in the Yellow River Delta consists primarily of saline–alkaline plants. *Tamarix chinensis*, a halophyte with salt-secretion capability, represents the most extensively distributed shrub in this region, owing to its high adaptability to soil salinity conditions. It is also a key species in the national “South Red North Willow” coastal wetland ecological restoration project and plays important roles in the ecological improvement and ecological restoration of coastal saline–alkaline soil [3,4]. In recent years, the Yellow River Delta has become an important ecological area for serving the major national Yellow River strategy. Research on the relationship between litter and soil in this area is increasing. At present, research on litter in the Yellow River Delta has primarily centered on the effects of different conditions, such as salinity and water submergence, on decomposition rates of halophytes such as *Phragmites australis*, *Suaeda salsa*, and *Spartina alterniflora* [5,6] and on the effects of plant litter on the quality of coastal saline soil under different shrub-grass patterns [7,8]. Litter decomposition by halophytes is complex under the influence of environmental change. The influence of different salinities on soil physicochemical properties is mostly inhibited, but *T. chinensis* litter may promote soil physicochemical properties in saline–alkaline soil suitable for soil salinity [9,10]. There is a lack of specific qualitative and quantitative research on the relationship between *T. chinensis* litter and soil under different salinities, which leads to an unclear effect of soil salinity on the decomposition rate of *T. chinensis* litter. Moreover, there remains a lack of systematic understanding of how soil salinity affects litter decomposition, soil nutrient content, and microbial community structure, as well as the mediating roles of bacteria and fungi in the feedback mechanisms linking soil salinity with litter decomposition and soil properties. This limits the ecological restoration and management of low-productivity *T. chinensis* forests in the Yellow River Delta.

In the ecosystem, litter is not only the main nutrient carrier but also the hub of material circulation [11,12]. The feedback process between litter and soil is obvious, and soil salinity is among the important environmental factors affecting litter decomposition [8,13]. Microorganisms are the main driving forces of litter decomposition [11,14], and soil salinity can affect litter decomposition rate by affecting soil microbial activity [15]. Strong soil salinity conditions reduce soil respiration and litter decomposition in the Athabasca Oil Sands Region [16]. An increase in salinity inhibits the activity of extracellular enzymes produced by soil microorganisms in freshwater wetlands, resulting in a decrease in organic matter decomposition [17]. Studies of the effects of saltwater intrusion on soil microbial organic carbon mineralization in tidal freshwater marshes have shown that high-salinity condition has a negative effect on soil physicochemical properties [18]. However, this finding may be due to the presence of litter, and the inhibition of salinity is alleviated to a certain extent because soil salinity can regulate the litter decomposition rate, thus affecting the nutrient release of the litter and subsequently affecting the soil structure, water retention, and other soil water physical characteristics, as well as soil nutrient content and microbial diversity [19]. Therefore, the specific interactions and mechanisms in this process need to be further studied. For example, under high-salinity conditions, as soil salinity increased, the toxicity of salinity inhibits the activity of enzymes and microorganisms and weakens the ability of microorganisms to degrade litter, thus inhibiting the decomposition of *Kandelia candel* litter [20,21,22]. Additionally, the presence of some salt-tolerant microorganisms can lead to an increase in soil salt content, which in turn promotes the decomposition of *Cyperus malaccensis* and *P. australis* in the wetlands of the Minjiang Estuary [5,23]. The rate of *P. australis* litter decomposition in the freshwater tidal wetland of the Hudson River was also studied. The results revealed that the change in soil salt content had no significant effect on the litter decomposition rate [24]. This is mainly because litter decomposition is also affected by other factors, such as temperature, humidity, litter quality, biological activity, and decomposition time [24]. Soil salinity has a certain degree of influence on litter decomposition rate, but results vary greatly across different research areas, habitats, and research objects. As such, the relationships between decomposition characteristics and soil physicochemical properties under different salinities and different *T. chinensis* litter decomposition degrees remain unclear. In particular, quantitative evidence for the interactions between litter decomposition and soil microbial communities is still lacking.

We previously studied the decomposition characteristics of *T. chinensis* litter under different salinity conditions and its impacts on soil physicochemical properties and enzyme activities. It was found that salinity-mediated *T. chinensis* litter decomposition in coastal saline–alkali soil of the Yellow River Delta increased soil enzyme activity [19]. However, previous studies have primarily focused on soil enzyme activity as a functional indicator. They have not yet elucidated whether changes in enzyme activity further select for and reshape specific microbial taxa, nor have they quantitatively partitioned the direct and indirect effects of soil salinity, litter decomposition, and soil physicochemical properties on microbial community structure. Based on this, we assume that this change will further screen out specific microbial groups. Therefore, according to the pre-established experimental design, following a 180-day experimental period, the litter decomposition rate, soil physicochemical properties, and microbial community structure were monitored and analyzed. High-throughput amplicon sequencing (e.g., 16S rRNA gene and ITS sequencing) was employed to characterize the composition and diversity of bacterial and fungal communities, and structural equation modeling (SEM) was conducted to quantify the coupled feedback mechanisms within the ‘soil salinity–litter decomposition–soil physicochemical properties–microbial community structure’ framework. The litter decomposition rate with different decomposition degrees under different soil salinity conditions was discussed; the primary objective was to elucidate how soil physicochemical properties and microbial community structure respond to varying salinity and litter decomposition. Furthermore, the study aimed to uncover the interactive relationships among soil salinity, litter decomposition rate, soil physicochemical properties, and microbial community structure. Nutrients are defined here as measured pools of inorganic and organic compounds that can support microbial growth and metabolism. The inorganic nutrient pool includes ammonium nitrogen (NH_4_^+^-N), nitrate nitrogen (NO_3_^−^-N), available phosphorus (AP), available potassium (AK), and related variables, whereas the organic nutrient pool includes total organic carbon (TOC), dissolved organic carbon (DOC), soil organic matter (SOM), and related variables. In this study, we primarily measured available nutrient contents because nutrient requirements are taxon-specific, and bulk nutrient pools do not necessarily represent the bioavailable fractions for all microorganisms. The findings are intended to inform management strategies for low-productivity *T. chinensis* forests in coastal saline–alkaline areas and to support the development of approaches for soil improvement in such environments.

## 2. Results

### 2.1. Characteristics of T. chinensis Litter Decomposition Rate

**Figure 1:**
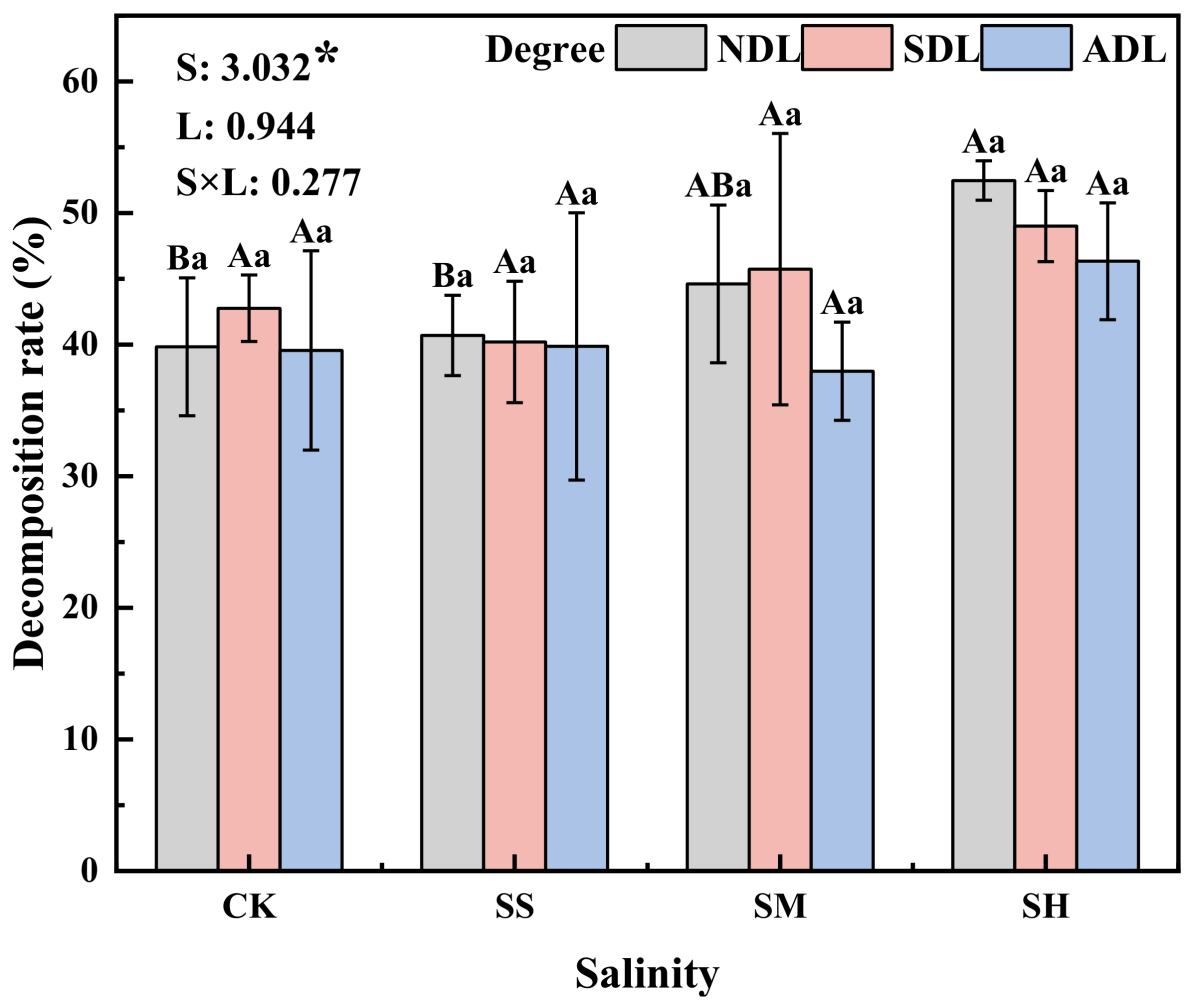
Decomposition rate of *Tamarix chinensis* litter under different salinity treatments. Notes: S: soil salt content; L: degree of litter decomposition; CK: control; SS: slight salinity; SM: moderate salinity; SH: high salinity; NDL: undecomposed; SDL: semidecomposed; ADL: already decomposed. * indicates significance at *p* < 0.05. Error bars represent standard deviations (SDs). Different uppercase letters indicate significant differences in various indicators among salinity treatments within the same litter decomposition degree (*p* < 0.05), whereas different lowercase letters indicate significant differences in various indicators among decomposition degrees within the same salinity treatment (*p* < 0.05).

Soil salinity exerted a significant influence on the decomposition rate of *T. chinensis* litter (*p* < 0.05), whereas litter decomposition degree and its interaction with salinity showed no significant influence (*p* > 0.05; Figure 1). Furthermore, for litter at the same decomposition degree, decomposition rates varied significantly across different salinity treatments (*p* < 0.05). As soil salinity increased, the litter decomposition rate tended to increase, by 5.05% and 21.01% under the moderate salinity (SM) and high salinity (SH) conditions, respectively, compared with that under the control (CK) condition. However, under the same soil salinity conditions, the litter decomposition rate did not differ significantly among the various decomposition degrees (*p* > 0.05).

### 2.2. Soil Physicochemical Properties

#### 2.2.1. Soil Bulk Density and Saturated Water Content

**Figure 2:**
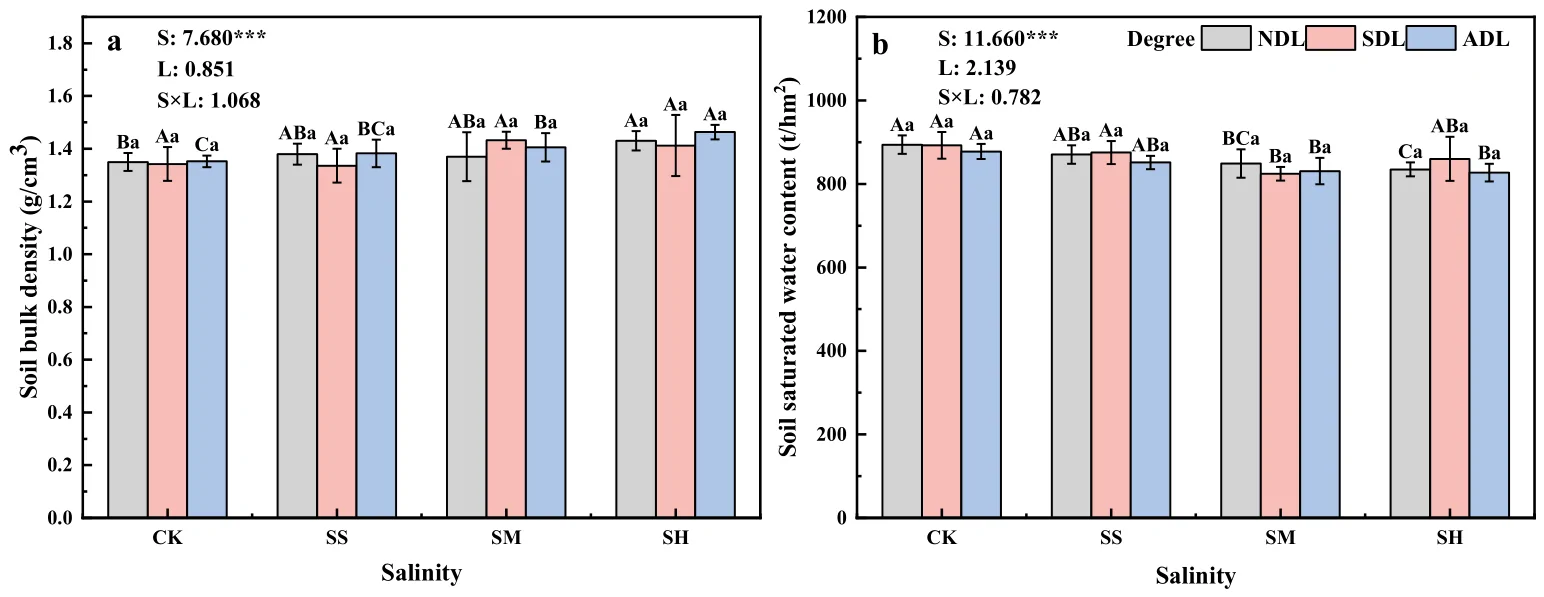
Soil bulk density and saturated water content under different salinity treatments. (**a**) Soil bulk density under different salinity treatments; (**b**) Soil saturated water content under different salinity treatments. Notes: S: soil salt content; L: degree of litter decomposition; CK: control; SS: slight salinity; SM: moderate salinity; SH: high salinity; NDL: undecomposed; SDL: semidecomposed; ADL: already decomposed. *** indicates significance at *p* < 0.001. Error bars represent standard deviations (SDs). Different uppercase letters indicate significant differences in various indicators among salinity treatments within the same litter decomposition degree (*p* < 0.05), whereas different lowercase letters indicate significant differences in various indicators among decomposition degrees within the same salinity treatment (*p* < 0.05).

Soil salinity had a highly significant influence on both soil bulk density and saturated water content (*p* < 0.001), whereas litter decomposition degree and its interaction with salinity showed no significant influence (*p* > 0.05; Figure 2). Moreover, for litter at the same decomposition degree, its effects on these two soil physical properties differed significantly across different salinity treatments (*p* < 0.05). As soil salinity increased, the soil bulk density increased significantly and was markedly greater under the SH condition than under the CK condition (*p* < 0.05), with an increase of 6.46%. In contrast, the soil saturated water content decreased significantly, with values under both the SM and SH conditions being significantly lower than those under the CK condition (*p* < 0.05), corresponding to reductions of 6.02% and 5.34%, respectively. Under the same soil salinity conditions, the soil physical properties did not differ significantly among the various decomposition degrees (*p* > 0.05).

#### 2.2.2. Soil Salt Content and Electrical Conductivity

**Figure 3:**
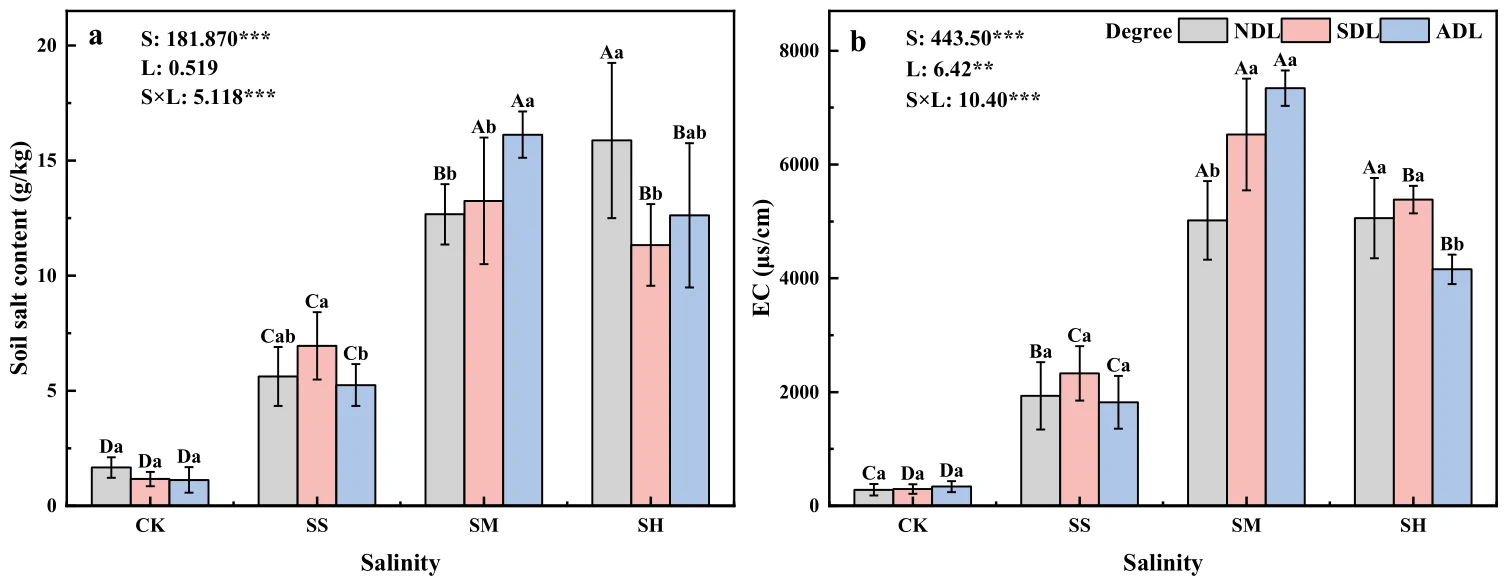
Soil salt content and electrical conductivity (EC) under different salinity treatments. (**a**) Soil salt content under different salinity treatments; (**b**) Soil electrical conductivity (EC) under different salinity treatments. Notes: S: soil salt content; L: degree of litter decomposition; CK: control; SS: slight salinity; SM: moderate salinity; SH: high salinity; NDL: undecomposed; SDL: semidecomposed; ADL: already decomposed. *** and ** indicate significance at *p* < 0.001 and *p* < 0.01, respectively. Error bars represent standard deviations (SDs). Different uppercase letters indicate significant differences in various indicators among salinity treatments within the same litter decomposition degree (*p* < 0.05), whereas different lowercase letters indicate significant differences in various indicators among decomposition degrees within the same salinity treatment (*p* < 0.05).

Soil salinity conditions, litter decomposition degree, and their interaction all exerted highly significant effects on both soil salt content and electrical conductivity (EC) (*p* < 0.01; Figure 3). Furthermore, for litter at the same decomposition degree, its influence on soil salt content varied significantly across the different salinity treatments (*p* < 0.05). As soil salinity increased, both soil salt content and EC first increased but then decreased, peaking under the SM condition. The specific order was SM > SH > slight salinity (SS) > CK, with values approximately 10.86, 9.50, and 4.46 times those of CK for soil salt content and 20.54, 15.88, and 6.62 times those for soil EC, respectively. Under the same soil salinity conditions, the litter decomposition degree significantly influenced both soil salt content and EC (*p* < 0.05). As litter decomposition degree increased, soil salt content and EC first increased but then decreased under the SS condition but gradually increased under the SM condition. Under the SM condition, values for both indicators were significantly higher in the already decomposed (ADL) than in the undecomposed (NDL), with increases of 27.30% and 46.8%, respectively (*p* < 0.05).

#### 2.2.3. Soil Available Nutrient Properties

**Figure 4:**
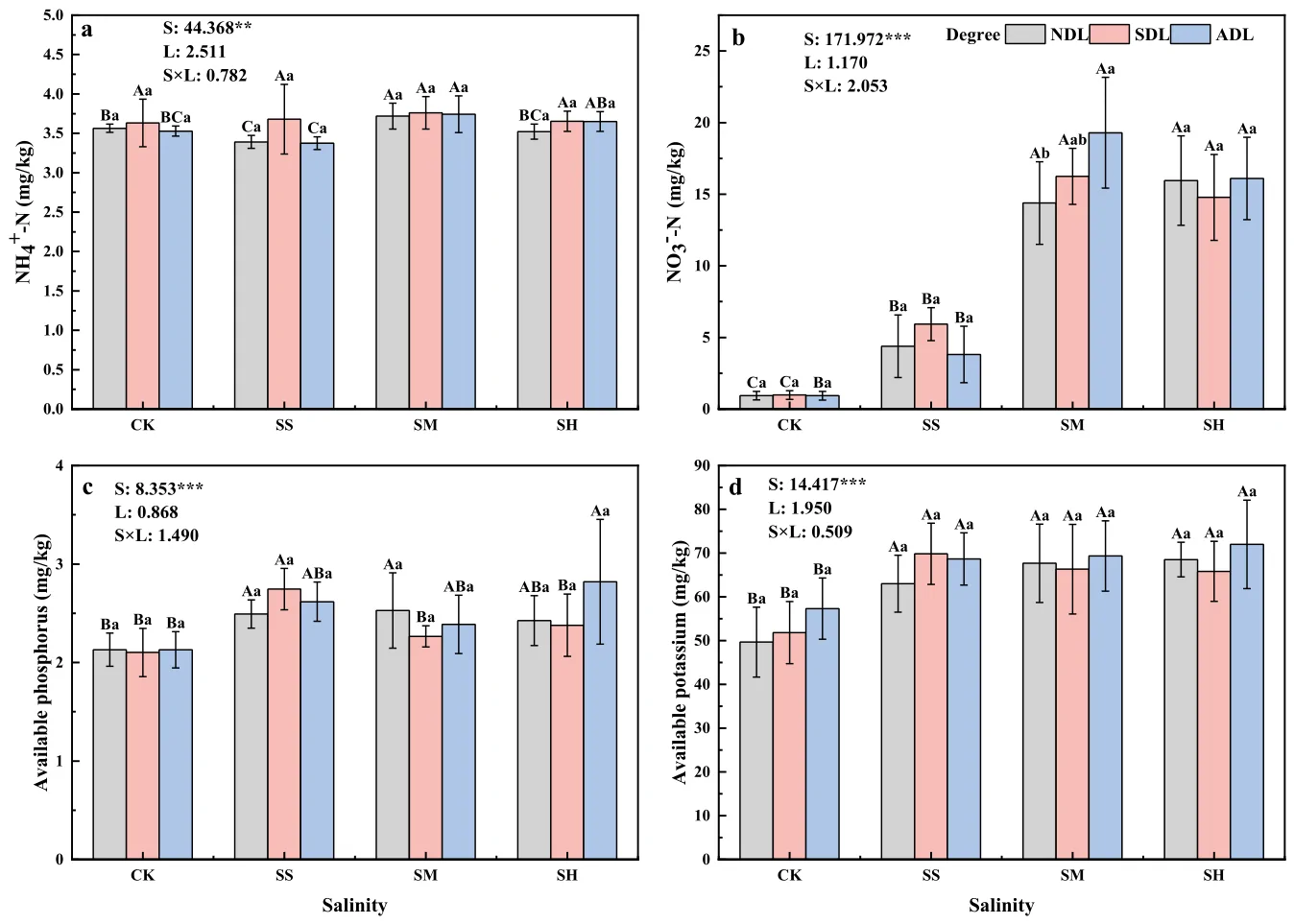
Soil available nutrient indicators under different salinity treatments. (**a**) Soil ammonium nitrogen (NH_4_^+^-N) content; (**b**) Soil nitrate nitrogen (NO_3_^−^-N) content; (**c**) Soil available phosphorus (AP) content; (**d**) Soil available potassium (AK) content. Notes: S: soil salt content; L: degree of litter decomposition; CK: control; SS: slight salinity; SM: moderate salinity; SH: high salinity; NDL: undecomposed; SDL: semidecomposed; ADL: already decomposed. *** and ** indicate significance at *p* < 0.001 and *p* < 0.01, respectively. Error bars represent standard deviations (SDs). Different uppercase letters indicate significant differences in various indicators among salinity treatments within the same litter decomposition degree (*p* < 0.05), whereas different lowercase letters indicate significant differences in various indicators among decomposition degrees within the same salinity treatment (*p* < 0.05).

Soil salinity had a highly significant influence on soil available nutrient contents (*p* < 0.001), whereas litter decomposition degree and its interaction with salinity showed no significant influence (*p* > 0.05; Figure 4). Moreover, for litter at the same decomposition degree, its impact on soil available nutrient contents differed significantly across different salinity treatments (*p* < 0.05). As soil salinity increased, the soil NH_4_^+^-N content peaked under the SM condition and was lowest under the SS condition. The soil NO_3_^−^-N content followed the order of SM > SH > SS > CK, whose values were approximately 17.54, 16.45, and 4.96 times greater, respectively, than those in the CK. The soil AP content under the SS, SM, and SH conditions was greater than that under the CK condition. The soil AK content gradually increased as soil salinity increased, by 26.86%, 28.02%, and 29.91% under the SS, SM, and SH conditions, respectively, compared with that under the CK. Under the same soil salinity conditions, litter at different degrees of decomposition had a significant effect on the soil NO_3_^−^-N content (*p* < 0.05), but no significant effects were observed on the other available nutrient indicators (*p* > 0.05). Under the SM condition, the NO_3_^−^-N content in the ADL was significantly greater than that in the NDL (*p* < 0.05), representing an increase of 34.11%.

### 2.3. Characteristics of Soil Microbial Diversity

#### 2.3.1. Soil Microbial Alpha Diversity

**Figure 5:**
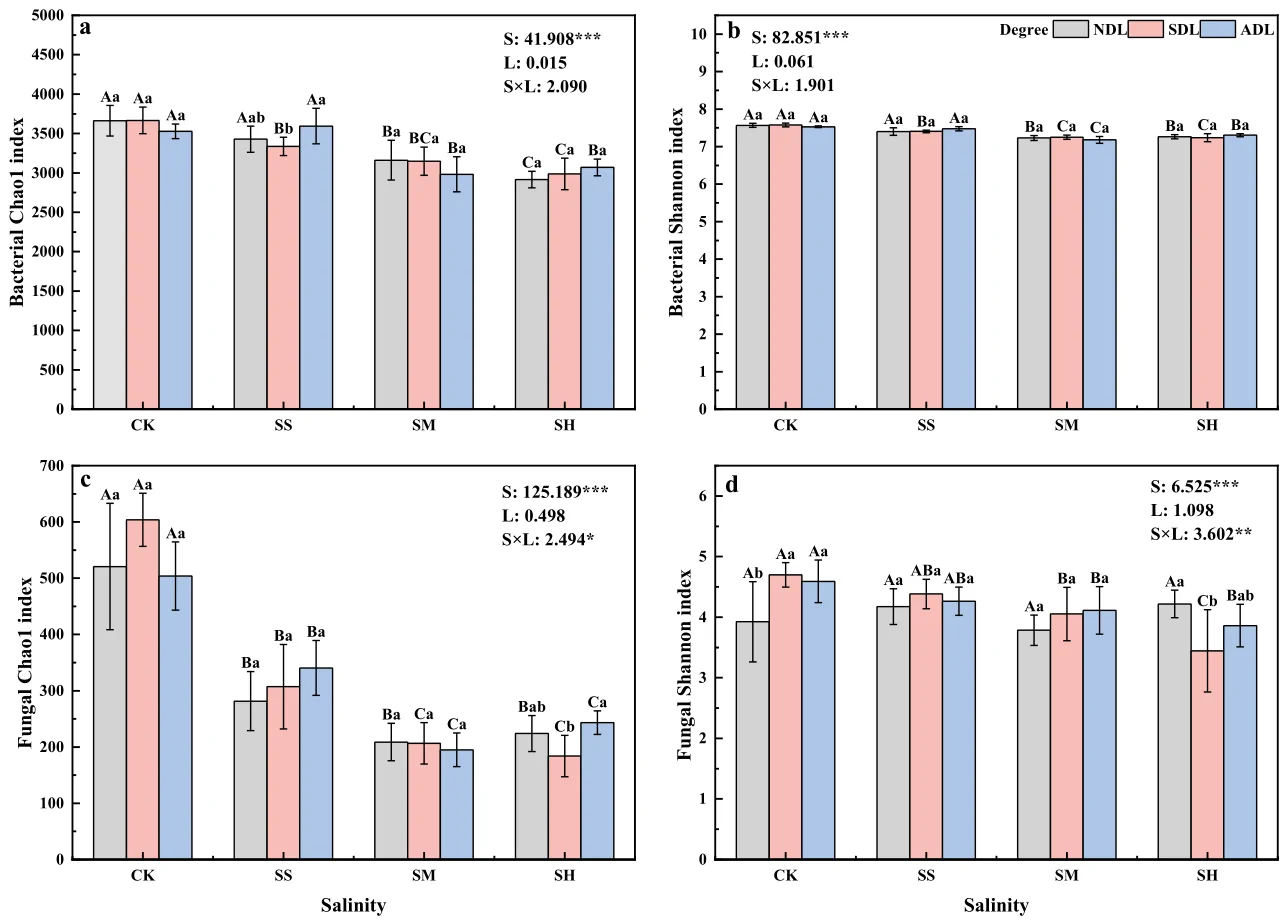
Soil microbial alpha diversity under different salinity treatments. (**a**) Soil bacterial Chao1 index; (**b**) Soil bacterial Shannon index; (**c**) Soil fungal Chao1 index; (**d**) Soil fungal Shannon index. Notes: S: soil salt content; L: degree of litter decomposition; CK: control; SS: slight salinity; SM: moderate salinity; SH: high salinity; NDL: undecomposed; SDL: semidecomposed; ADL: already decomposed. ***, ** and * indicate significance at *p* < 0.001, *p* < 0.01, and *p* < 0.05, respectively. Error bars represent standard deviations (SDs). Different uppercase letters indicate significant differences in various indicators among salinity treatments within the same litter decomposition degree (*p* < 0.05), whereas different lowercase letters indicate significant differences in various indicators among decomposition degrees within the same salinity treatment (*p* < 0.05).

Soil salinity had a highly significant influence on both bacterial and fungal alpha diversity (*p* < 0.001). Soil salinity had significant influence on fungal alpha diversity (*p* < 0.05) and its interaction with decomposition degree (*p* < 0.01), but not to decomposition degree alone (*p* > 0.05; Figure 5). Moreover, for litter at the same decomposition degree, its impact on microbial alpha diversity differed significantly across different salinity treatments (*p* < 0.05). As soil salinity increased, both the bacterial Chao1 index and the bacterial Shannon index tended to decrease. Under both the SM and SH conditions, these indices were significantly lower than those under the CK condition (*p* < 0.05), with reductions of 14.42% and 17.37% for the Chao1 index and 4.46% and 3.82% for the Shannon index, respectively. Similarly, both the fungal Chao1 index and the fungal Shannon index tended to decrease and were significantly lower under the SM and SH conditions than under the CK condition (*p* < 0.05), with decreases of 62.52% and 59.99% for the Chao1 index, and 9.57% and 12.79% for the Shannon index, respectively.

Under the same soil salinity conditions, different degrees of decomposition significantly affected the bacterial Chao1 index, the fungal Chao1 index, and the fungal Shannon index (*p* < 0.05), but no significant effect was observed on the bacterial Shannon index (*p* > 0.05). As litter decomposition degree increased, the bacterial and fungal Chao1 indices first decreased but then increased under the SS and SH conditions, respectively. The fungal Shannon index significantly decreased under the SH condition, with the semidecomposed (SDL) being 18.35% lower than the NDL.

#### 2.3.2. Soil Microbial Beta Diversity

**Figure 6:**
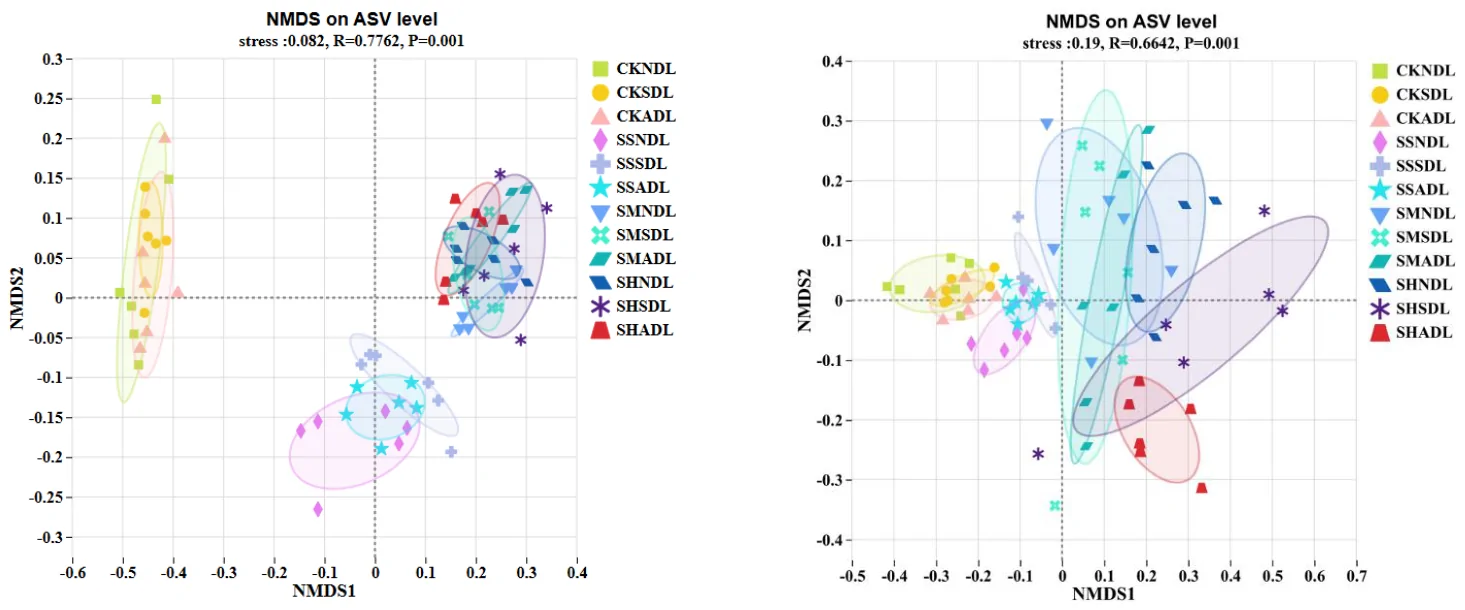
Soil microbial beta diversity under different salinity treatments. Notes: CK: control; SS: slight salinity; SM: moderate salinity; SH: high salinity; NDL: undecomposed; SDL: semidecomposed; ADL: already decomposed.

Non-metric multidimensional scaling (NMDS) analysis was further conducted to investigate the effects of litter at different degrees of decomposition on soil microbial community diversity under varying salinity conditions (Figure 6). The soil bacterial and fungal community structures under the SS, SM, and SH conditions differed from those under the CK condition. Specifically, the bacterial community structures under the SM and SH conditions strongly overlapped, whereas the fungal community structures partially overlapped. Under the same soil salinity conditions, no significant differences (*p* > 0.05) in the effects of litter at different degrees of decomposition on soil microbial community diversity were detected. These findings indicate that changes in soil salinity conditions have a stronger influence on the soil microbial community than does the degree of litter decomposition.

### 2.4. Soil Microbial Species Composition

#### 2.4.1. Venn Diagram of Soil Microbial Amplicon Sequence Variants (ASVs)

**Figure 7:**
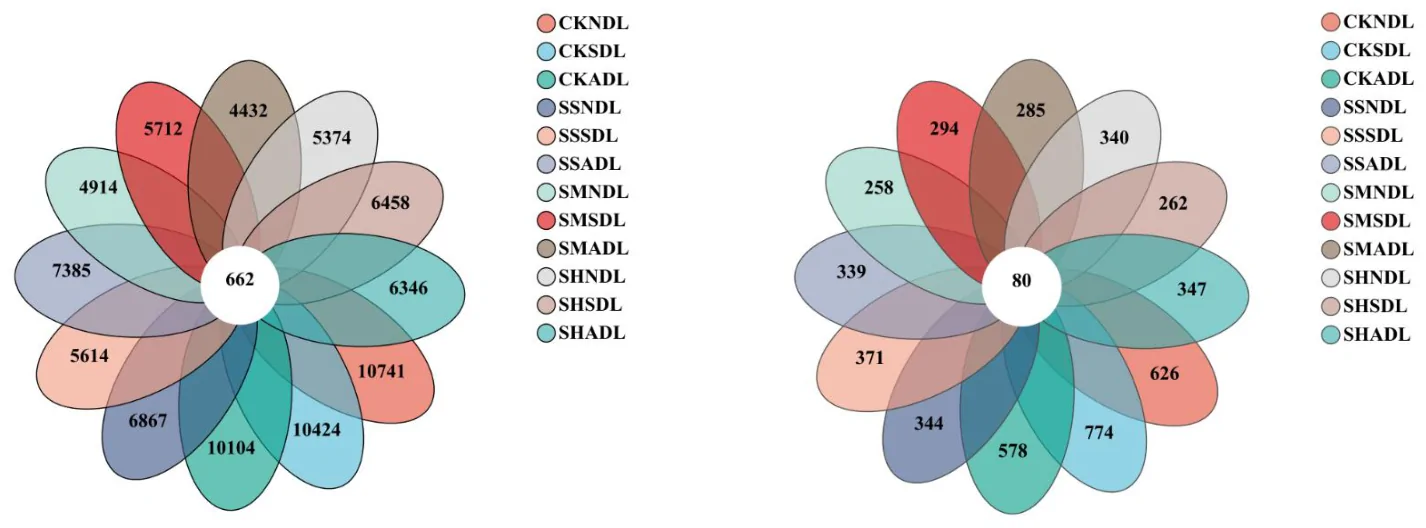
Venn diagram analysis of soil microbial amplicon sequence variants (ASVs) under different salinity treatments. Notes: CK: control; SS: slight salinity; SM: moderate salinity; SH: high salinity; NDL: undecomposed; SDL: semidecomposed; ADL: already decomposed.

As shown in Figure 7, 662 of ASVs were shared by the soil bacterial communities across all the different salinity conditions and degrees of litter decomposition, while that for the fungal communities was 80. Under the SS, SM, and SH conditions, the numbers of unique ASVs for both the soil bacterial and fungal communities were lower than those under the CK condition. Moreover, the numbers of unique ASVs under the SM and SH conditions were lower than those under the SS condition, indicating that soil salinity reduces the number of unique microbial ASVs in coastal saline‒alkaline soil. No significant differences (*p* < 0.05) were detected in the numbers of bacterial and fungal community ASVs among the different degrees of litter decomposition under the same salinity conditions. This lack of significant difference may be attributed to the relatively short duration of the *T. chinensis* litter decomposition process in this study.

#### 2.4.2. Soil Microbial Relative Abundance

**Figure 8:**
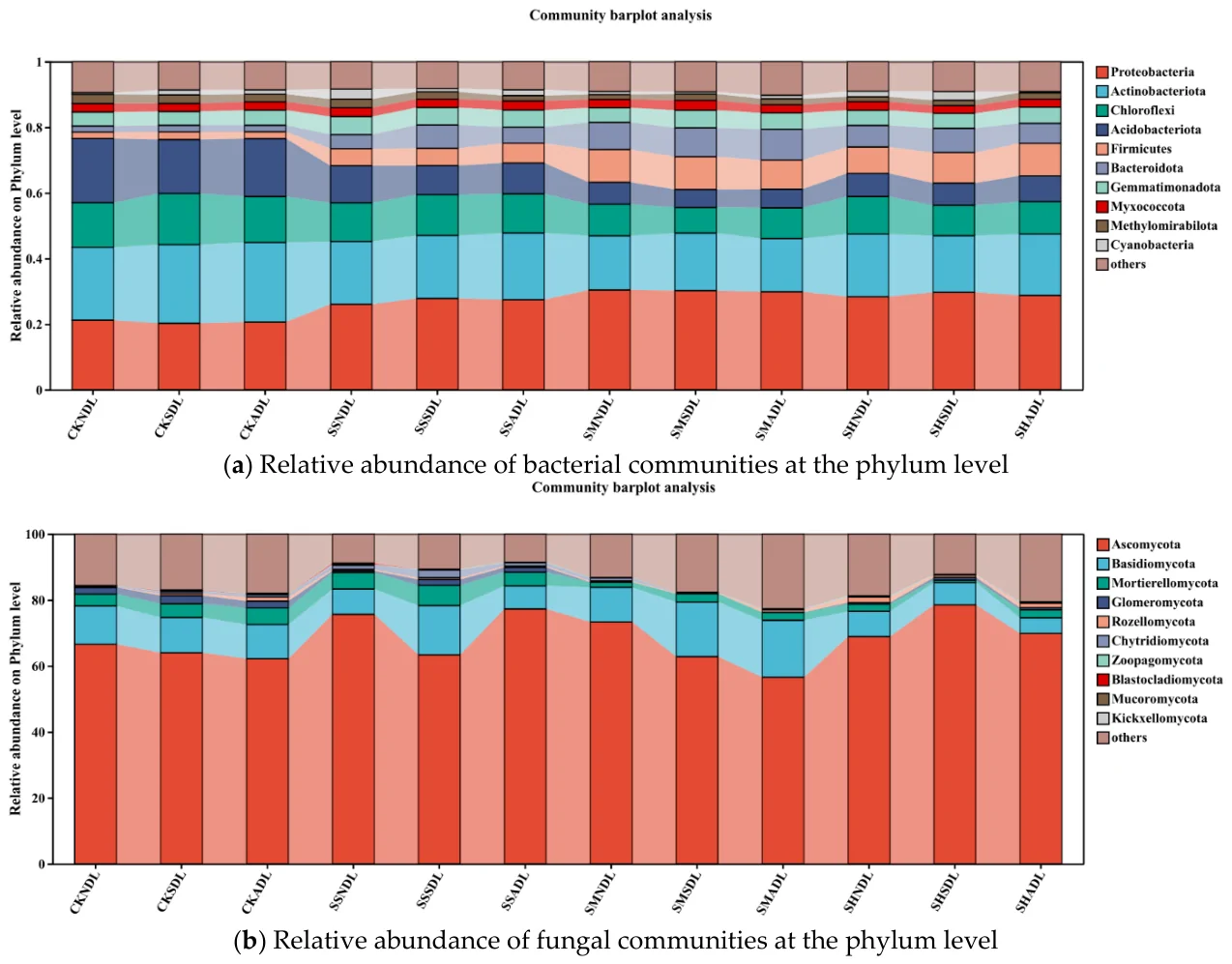
Relative abundance of soil microbiota at the phylum level under different salinity treatments. Notes: CK: control; SS: slight salinity; SM: moderate salinity; SH: high salinity; NDL: undecomposed; SDL: semidecomposed; ADL: already decomposed.

The relative abundances of soil bacterial and fungal communities at the phylum level are shown in Figure 8 under different salinity conditions and degrees of litter decomposition. The top 10 bacterial phyla in terms of relative abundance were Proteobacteria, Actinobacteriota, Chloroflexi, Acidobacteriota, Firmicutes, Bacteroidota, Gemmatimonadota, Myxococcota, Methylomirabilota, and Cyanobacteria. Collectively, these phyla accounted for 89.73% to 91.82% of the total bacterial abundance across all treatments. Compared with the CK condition, the different soil salinity conditions significantly increased the relative abundances of Proteobacteria, Firmicutes, and Bacteroidota. Under the same soil salinity conditions, no significant differences were observed in the relative abundances of soil bacterial communities at the phylum level among the different degrees of litter decomposition (*p* > 0.05).

The top 10 fungal phyla in terms of relative abundance were Ascomycota, Basidiomycota, Mortierellomycota, Glomeromycota, Rozellomycota, Chytridiomycota, Zoopagomycota, Blastocladiomycota, Mucoromycota, and Kickxellomycota. Collectively, these phyla accounted for 77.30% to 91.47% of the total fungal abundance across all treatments. Under the same soil salinity conditions, the relative abundance of soil fungal communities at the phylum level did not consistently change across the different degrees of litter decomposition.

#### 2.4.3. Heatmap Analysis of the Soil Microbial Community

**Figure 9:**
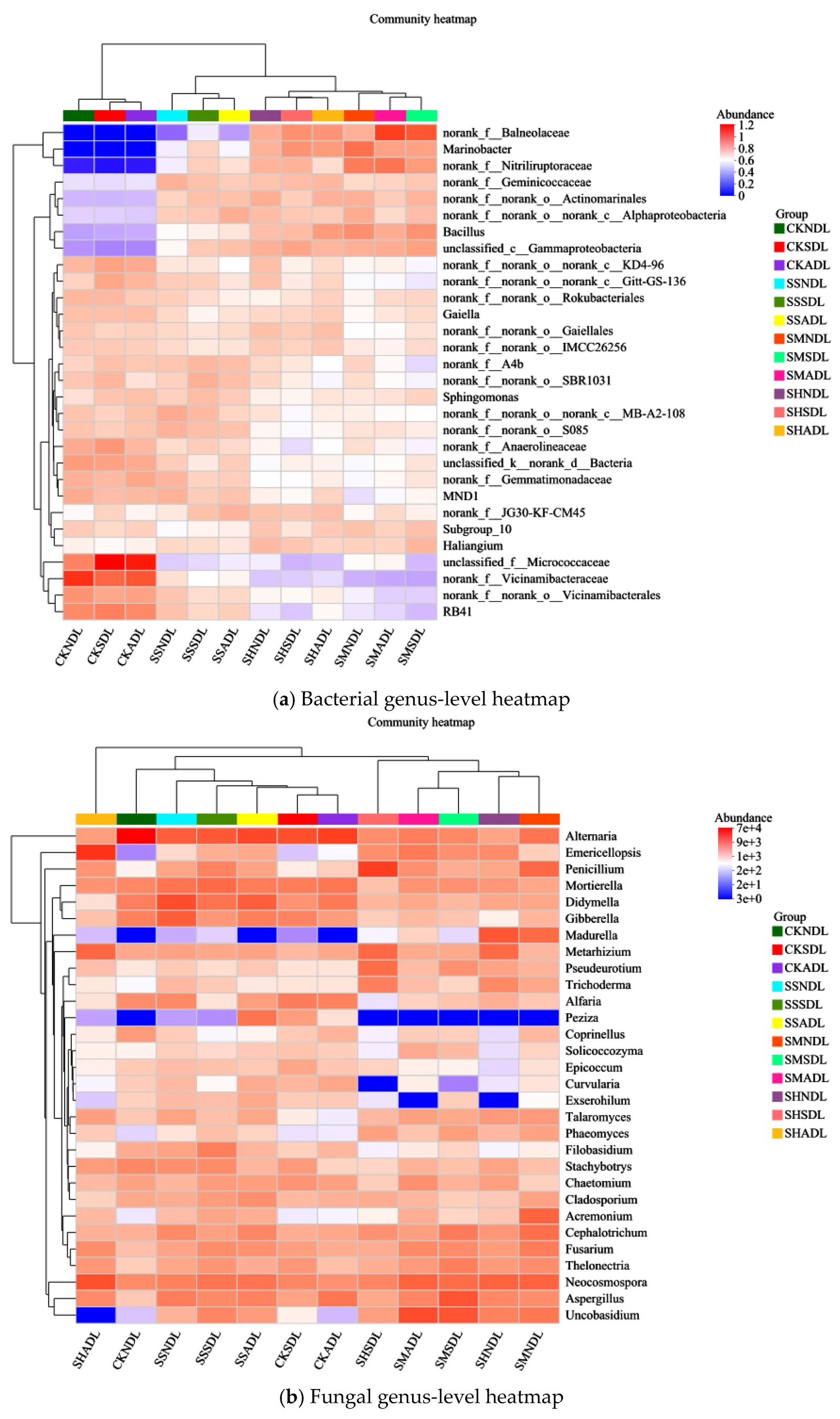
Heatmaps of species composition at the genus level of soil microbiota under different salinity treatments. Notes: CK: control; SS: slight salinity; SM: moderate salinity; SH: high salinity; NDL: undecomposed; SDL: semidecomposed; ADL: already decomposed.

A heatmap illustrating the results of the cluster analysis of soil bacterial and fungal communities at the genus level under different salinity conditions and degrees of litter decomposition is presented in Figure 9. Thirty bacterial genera and thirty fungal genera were selected from each sample for analysis. Significant differences (*p* < 0.05) in the composition of some soil bacterial genera were detected between the SS, SM, and SH conditions and the CK condition. Furthermore, bacterial genera composition was notably similar between the SM and SH conditions. For instance, genera such as *Marinobacter*, *Bacillus*, and some unclassified bacterial genera were significantly or nonsignificantly enriched under various salinity conditions but were relatively less abundant under the CK condition. As soil salinity increased, most bacterial genera remained either dominant or nonsignificantly enriched. Some genera, such as *Marinobacter* and *Bacillus*, shifted from being less abundant to being significantly enriched, whereas others, such as RB41, transitioned from being significantly enriched to becoming less abundant.

The results of the fungal cluster analysis at the genus level revealed significant differences (*p* < 0.05) among the soil fungal communities under different salinity conditions. The composition of fungal genera was relatively similar between the SM and SH conditions, and between the SS and CK conditions. For example, the genus *Madurella* was either nonsignificantly enriched or dominant under the SM and SH conditions but was less abundant under the CK and SS conditions. As soil salinity increased, the abundances of genera such as *Mortierella*, *Didymella*, *Gibberella*, *Pseudeurotium*, and *Trichoderma* remained relatively dominant. Specifically, *Mortierella*, *Didymella*, and *Gibberella* were significantly enriched under the CK and SS conditions, whereas *Pseudeurotium* and *Trichoderma* were significantly enriched under the SM and SH conditions. Conversely, *Peziza* was less abundant under the SM and SH conditions but was either significantly enriched or relatively less abundant under the CK and SS conditions. Genera such as *Epicoccum*, *Curvularia*, and *Exserohilum* were either nonsignificantly enriched or less abundant under the SM and SH conditions but were relatively dominant under the CK and SS conditions.

### 2.5. Relationships Among T. chinensis Litter Decomposition, Soil Environmental Factors, and Microbial Communities

#### 2.5.1. Mantel Test Network Heatmap Among Environmental Factors

**Figure 10:**
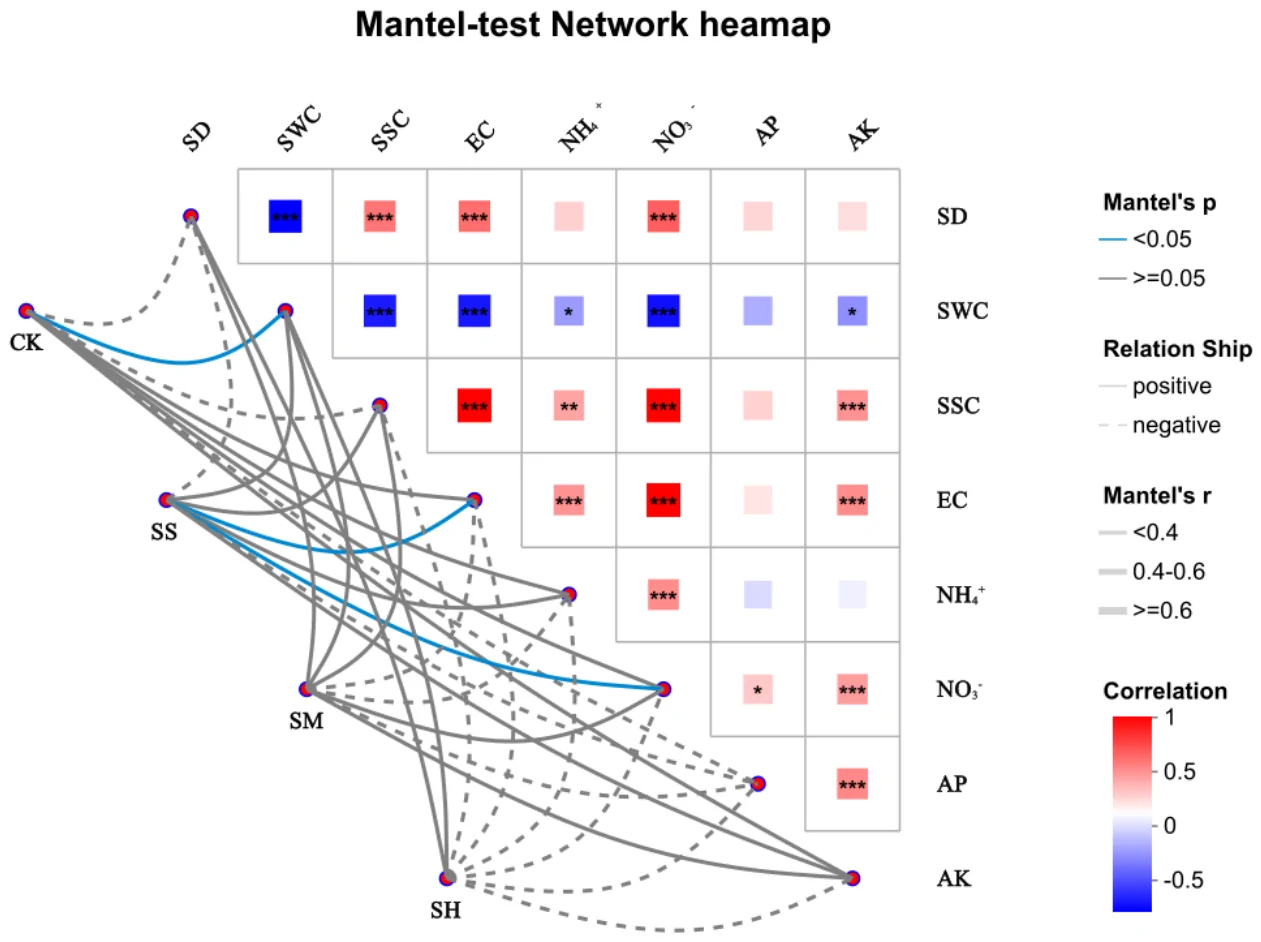
Correlation analysis of soil environmental factors. Notes: CK: control; SS: slight salinity; SM: moderate salinity; SH: high salinity; SD: soil bulk density; SWC: soil saturated water content; SSC: soil salt content; AP: available phosphorus; AK: available potassium. ***, ** and * indicate significance at *p* < 0.001, *p* < 0.01, and *p* < 0.05, respectively.

The results of the Mantel test (Figure 10) revealed that SM and SH were positively correlated with soil bulk density, indicating that increased salinity exacerbates soil compaction. CK, SS, SM, and SH conditions were positively correlated with the soil saturated water content but negatively correlated with the soil AP content. SH was negatively correlated with soil salt content, suggesting that the decomposition of *T. chinensis* litter may alleviate soil salinity under high-salinity conditions. SM and SH were negatively correlated with the soil NH_4_^+^-N content, whereas SH was negatively correlated with the soil AK content, implying that soil salinity may inhibit the effectiveness of nutrients but not significantly.

Further analysis of the specific relationships among soil environmental factors was conducted using Spearman correlation heatmaps (Figure 10). Soil bulk density was strongly positively correlated (*p* < 0.01) with soil salt content, EC, and NO_3_^−^-N content. The soil saturated water content was negatively correlated with all the measured indicators. Soil salt content and EC were significantly (*p* < 0.05) or highly significantly (*p* < 0.01) positively correlated with the NH_4_^+^-N, NO_3_^−^-N, and AK contents. The NH_4_^+^-N content was highly significantly positively correlated (*p* < 0.01) with the NO_3_^−^-N content, and significant (*p* < 0.05) or highly significant (*p* < 0.01) positive correlations were detected among the NO_3_^−^-N, AP, and AK contents, indicating significant positive coupling relationships among soil nutrients.

#### 2.5.2. Analysis of the Structural Equation Modeling Results

**Figure 11:**
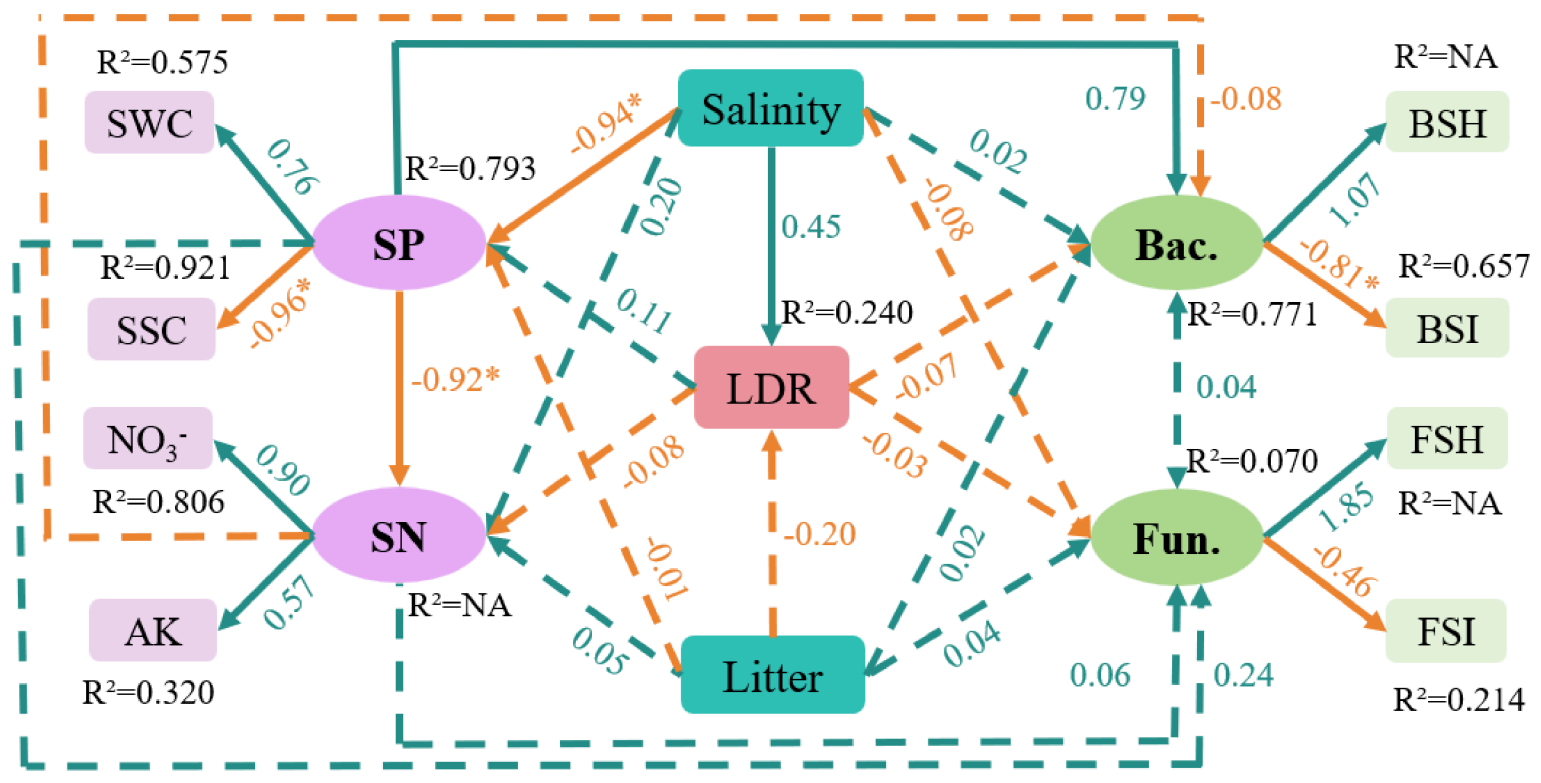
Structural equation model illustrating the relationships among the *T. chinensis* litter decomposition rate, soil environmental factors, and microbial communities. Notes: Salinity: soil salinity; Litter: degree of litter decomposition; LDR: litter decomposition rate; SP: soil physical indicators; SN: soil available nutrient content; SWC: soil saturated water content; SSC: soil salt content; AK: soil available potassium; BSH: bacterial Shannon index; BSI: bacterial Simpson index; FSH: fungal Shannon index; FSI: fungal Simpson index. * indicates significance at *p* < 0.05. The number on the arrow is the standardized path coefficient. The solid line represents the relationships between variables (*p* < 0.05), and the dotted line represents the relationships between variables that are not significant (*p* > 0.05). Green represents a positive effect and orange represents a negative effect.

Based on correlation and Mantel test analyses between litter and soil, variables exhibiting significant correlations were selected, and a structural equation modeling (SEM) was constructed using R (version 4.3.2). The SEM was further employed to explore the relationships among *T. chinensis* litter, soil environmental factors, and microbial communities. The model demonstrated a good fit, with a goodness-of-fit index (GFI) of 0.957 and a comparative fit index (CFI) of 0.965 (Figure 11). The SEM results indicated that soil salinity had a significant effect on the litter decomposition rate and soil physical properties (*p* < 0.05), with path coefficients of 0.45 and −0.94, respectively. Soil physical properties had a significant negative effect on soil available nutrient contents (*p* < 0.05; path coefficient = −0.92) and a significant positive effect on bacterial diversity (*p* < 0.05; path coefficient = 0.79). The degree of litter decomposition did not significantly affect the litter decomposition rate, soil physicochemical properties, or microbial diversity (*p* > 0.05). This may be primarily attributed to the dominant role of soil salinity. In the saline–alkaline environment of the Yellow River Delta, the effects of soil salinity on soil properties were stronger than the substrate quality differences represented by the degree of litter decomposition. This may also be associated with the higher lignin content and slower decomposition of *T. chinensis* litter, such that the effects of litter on soil microbial communities may become significant only during the later stages of decomposition. In this study, compared with soil available nutrients, soil physical properties had a more pronounced influence on microbial diversity.

## 3. Discussion

### 3.1. Response of the T. chinensis Litter Decomposition Rate to Different Soil Salinity Conditions

In the ecological restoration of coastal saline–alkaline soils in the Yellow River Delta, *T. chinensis* is commonly used as a pioneer restoration species [3,25]. The Yellow River Delta is a typical coastal saline-alkaline ecosystem, formed by long-term sediment deposition and frequent seawater intrusion. Sediments in the lower reaches of the Yellow River Basin are mainly derived from the middle reaches, and reservoir construction over recent decades has significantly altered downstream sediment transport. These depositional processes, together with tidal scouring and shallow groundwater evaporation, collectively contribute to the accumulation of soluble salts in deltaic soils, in which Na^+^ is the dominant cation and Cl^−^ and SO_4_^2−^ are the major anions. These regional geological and hydrological conditions have fundamentally shaped the salinity gradients, nutrient status, and microbial habitats examined in this study [26]. In this study, the decomposition rate of *T. chinensis* litter increased as soil salinity increased, indirectly indicating an increased rate of organic matter mineralization. This process may promote the rapid release of nutrients such as N, P, and K in the form of inorganic salts [27], thereby alleviating nutrient limitations in saline‒alkaline soils to a certain extent. SEM revealed a key pathway through which soil salinity promotes the decomposition of *T. chinensis* litter, with a path coefficient of 0.45. This aligns with the findings of Zhai et al. [5], who reported significantly higher decomposition rates for the litter of the halophytes *P. australis* and *S. salsa* under higher salinity conditions in the Yellow River Delta wetlands, with decomposition rates positively correlated with salinity. This phenomenon may be attributed to the adaptation of *T. chinensis*, *P. australis*, and *S. salsa* to high-salinity environments, where indigenous microorganisms have evolved a degree of salt tolerance [5,28]. These salt-tolerant microbes efficiently utilize relatively enriched available nutrients. Their efficient enzymatic systems and metabolic activity largely overcome the constraints imposed by deteriorating soil physical structure. This phenomenon ultimately manifests as an accelerated litter decomposition rate as soil salinity increased. Therefore, the influence of soil salinity on litter decomposition rate may be mediated by soil microorganisms [29]. The accelerated decomposition of *T. chinensis* litter under high-salinity conditions represents a unique survival strategy that evolved under long-term salt stress. It is precisely the dominant microbial groups (e.g., Proteobacteria) that persist under salinity selection that also promote litter decomposition, potentially revealing the unique patterns of material cycling in saline‒alkaline ecosystems. In contrast, the litter decomposition rate of a dominant species in the Athabasca Oil Sands Region was higher under light salinity and high-salinity conditions but lower under moderate- and extremely high-salinity conditions [16]. This discrepancy might be attributed to a decline in soil microbial biomass or activity under high-salinity [30,31]. Under high-salinity stress, microorganisms can tolerate and adapt to a certain range of ECs by absorbing salinity or synthesizing compatible solutes to prevent water loss and maintain cell turgor and metabolism [32]. Therefore, microbially mediated litter decomposition may be subject to constraints along different soil salinity gradients. Decomposition may be promoted within an optimal salinity range. However, salinity itself may not have a strong direct effect on litter decomposition during coastal forest retreat. Instead, temperature and moisture can increase decomposition by increasing bacterial and fungal respiration, whereas decomposition is likely inhibited under very high salinity levels [33]. At Phillips Creek, increasing temperature elevated the leaf litter decomposition rate independently of substrate quality [33]. This suggests that *T. chinensis* litter decomposition may also be influenced by temperature. Previous findings from our research group indicated that soil water content increases significantly as soil salinity increased [19]. This increase in moisture can increase fungal and microbial productivity, potentially contributing to an increased decomposition rate of *T. chinensis* litter under high-salinity conditions [34].

### 3.2. Effects of T. chinensis Litter Decomposition on Soil Physicochemical Properties Under Different Soil Salinity Conditions

Accelerated *T. chinensis* litter decomposition promotes organic matter mineralization and nutrient release, significantly increasing soil nutrient availability in saline‒alkaline environments in the short term and thereby contributing to faster soil fertility improvement [35]. In this study, the soil salt content and EC initially increased but then decreased as soil salinity increased, specifically in the order SM > SH > SS > CK, and were positively correlated with NH_4_^+^-N, NO_3_^−^-N, and AK contents. A negative correlation was observed between high-salinity conditions and soil salt content. This suggests that the optimal salinity range for *T. chinensis* litter decomposition lies within high-salinity environments, where salt-tolerant microorganisms are selected for and sensitive taxa are eliminated. As soil salinity increased, soil bulk density increased significantly, whereas soil saturated water content decreased significantly. These changes indicate reduced soil porosity, impaired aeration, and diminished water-holding capacity. Soil bulk density was significantly positively correlated with soil salinity and significantly negatively correlated with soil saturated water content. Moderate- and high-salinity conditions were positively correlated with soil bulk density, indicating that increased salinity exacerbates soil compaction. This phenomenon is likely due to salt ions aggravating soil compaction, disrupting soil capillary structure, weakening water-holding capacity, and restricting the diffusion of water and oxygen [36]. These findings align with observations in the Yellow River Delta coastal wetlands, where soil respiration rates generally decrease with increasing salinity [2]. Soil bulk density increases significantly along a salinization gradient [37]. Similar trends have been reported in studies on brackish water irrigation [38]. In contrast to the deterioration of physical structure, the soil available nutrient contents under slight-, moderate-, and high-salinity conditions were significantly greater than those under the CK condition, providing relatively abundant nutrient substrates for microorganisms. Positive correlations were observed among soil NO_3_^−^-N, AP, and AK contents. This may be attributed to soil salinity accelerating *T. chinensis* litter decomposition, thereby increasing soil nutrient levels. These results are consistent with findings that litter decomposition improves soil nutrient conditions in desert-oasis ecotones [39] and that the addition of litter from *Phoebe bournei*, *Schima superba*, and *Cunninghamia lanceolata* significantly increases soil alkaline-hydrolyzable N and AP contents in artificial forests in hilly areas of south-central Jiangxi [40]. Soil NH_4_^+^-N content was highest under moderate-salinity conditions and lowest under slight-salinity conditions. The activities of β-glucosidase and leucine aminopeptidase secreted by Proteobacteria in high-salinity environments increased, directly accelerating the release of C and N. Saline‒alkali habitats often face P limitation, as calcium-phosphate precipitation in high-pH saline soils reduces P availability [41]. However, in this study, the soil AP content under slight-, moderate-, and high-salinity conditions was greater than that under the CK condition. This may be due to increased salinity promoting mineral dissolution, as Na^+^ displaces adsorbed P, thereby enhancing P availability in the short term [42]. Research from the Changling Ecological Research Station for Grassland Farming, which is located in the south of the Songnen Plain of Northeast China, also revealed correlations among soil nutrients in cropland, grassland, and successional regenerated grassland [43]. Similarly, this study revealed significant positive coupling relationships among soil nutrients, as litter decomposition can simultaneously release C, N, P, and K to improve soil nutrient status [44].

### 3.3. Effects of T. chinensis Litter Decomposition on the Soil Microbial Community Structure Under Different Salinity Conditions

Soil microbial activity directly influences ecosystem stability and fertility [45]. Microbial nutrient requirements are functionally and phylogenetically diverse. Heterotrophic bacteria and fungi depend largely on organic carbon. Nitrifying microorganisms use NH_4_^+^ as electron donors, whereas denitrifiers use NO_3_^−^ as electron acceptors. Therefore, the same bulk nutrient pool may be limiting for one microbial group but non-limiting for another. In the Yellow River Delta, salinity, pH, and moisture further modulate nutrient bioavailability. Accordingly, we interpret nutrient variables as potential resource availability. In this study, we reported that the Chao1 index (richness), Shannon‒Wiener index (diversity), and number of ASVs for both soil bacterial and fungal communities significantly decreased as soil salinity increased. These findings align with observations across a broad salinity gradient (from coastal to inland China, 0.06 to 116 mS/cm), where soil salinization has been shown to suppress microbial diversity, likely because of a filtering effect that favors halotolerant and halophilic microorganisms while disadvantaging salt-sensitive taxa, subsequently altering the microbial community structure [46]. However, a contrasting study in the Hetao Irrigation District of Inner Mongolia reported higher species richness and diversity of rhizosphere soil bacteria in *Suaeda glauca* communities under high saline‒alkali conditions than under slight- and moderate-salinity conditions [47]. These findings suggest that many sensitive microbial species have been eliminated in the screening of saline–alkaline environments in the Yellow River Delta and that the community composition is highly specialized. Soil physicochemical properties affected the microbial community structure after more than 40 years of natural restoration and afforestation in karst fault basins in China [48]. However, SEM in this study revealed a significant positive effect of soil physical properties on bacterial diversity (*p* < 0.05), with a path coefficient of 0.79. Notably, the influence of soil physical properties on microbial diversity was more pronounced than that of soil available nutrients. This discrepancy may be attributed to the relatively short duration of *T. chinensis* litter decomposition in our study. The nutrients released from litter decomposition may not have been fully assimilated by the soil matrix, thus exerting a limited effect on microbial diversity within the observed timeframe. Furthermore, the beta diversity of the microbial communities under slight-, moderate-, and high-salinity conditions significantly differed from that of the CK condition. Strong overlap was observed between communities under moderate- and high-salinity conditions, indirectly indicating that the salinity gradient resulted in the formation of a highly similar, salinity-adapted core microbial community structure (e.g., dominated by Proteobacteria). This convergence further suggests that only a subset of species may be able to adapt to high-salinity habitats, leading to increased community similarity under highsalinity conditions.

Members of the phylum Proteobacteria secrete extracellular enzymes that degrade labile organic matter, driving the rapid early stage decomposition of litter [49,50]. Bacteria belonging to the phylum Firmicutes possess cell walls with a high peptidoglycan content and can form endospores, conferring resistance to desiccation and extreme environments [50,51]. Bacteroidota are multishaped rods with the metabolic ability to degrade complex polysaccharides [50]. In this study, compared with the CK treatment, the slight-, moderate-, and high-salinity treatments significantly increased the relative abundance of Proteobacteria, Firmicutes, and Bacteroidota in the soil. Notably, Proteobacteria was dominant in saline–alkaline soils and was the most abundant bacterial phylum in this study. Fungi of the phylum Ascomycota are characterized by the formation of an ascus during sexual reproduction. This phylum is ubiquitous in nature and plays major roles in ecological processes such as the global carbon cycle, litter decomposition, and organic matter mineralization [50,51]. Compared with the CK treatment, the slight and high-salinity treatments significantly increased the relative abundance of Ascomycota, which was the dominant fungal phylum in this study. These findings indirectly indicate that as soil salinity increased, microbial taxa adapted to low-salinity environments are suppressed, whereas highly specialized halotolerant and halophilic phyla gradually become dominant, soil enzyme activities were also higher under these salinity conditions, thereby driving litter decomposition. This finding aligns with observations from the Hetao Plain, which is located along the middle–upper reaches of the Yellow River, within the transitional area between the Yellow River alluvial plain and the Ho-Lan Mountain flood plain, where various archaeal groups were found in highly saline–alkaline habitats [52], suggesting that soil salinity-driven microbial niche differentiation is obvious. Tracking changes at the genus level revealed that *Marinobacter* belongs to γ-Proteobacteria and *Alteromonas*, with moderate halophilicity [53]. *Bacillus* is a Gram-positive rod-shaped bacterium that is strongly resistant to physicochemical factors such as high temperature, dryness, and high salinity. Endospores form under conditions such as nutrient deprivation or drought and revert to vegetative bodies when conditions are suitable [54]. These results revealed that, compared with those in the CK treatment, the abundances of *Marinobacter*, *Bacillus*, and several unclassified bacterial genera were significantly greater under slight-, moderate-, and high-salinity conditions. Furthermore, the compositional profiles of the bacterial genera were notably similar between the moderate- and high-salinity conditions. The genus *Pseudeurotium*, a member of the phylum Ascomycota, is widely distributed in soil, wetlands, and plant endophytic environments and is involved in organic matter decomposition [55]. Species of the genus *Trichoderma* are commonly found in saline–alkaline soils and exhibit high decomposition efficiency. Certain species (e.g., *Tricoderma asperellum*) can ameliorate saline‒alkali soil by regulating the soil pH and increasing water retention, organic matter, and the availability of N, P, and K [56]. In this study, *Pseudeurotium* and *Trichoderma* were significantly enriched under the SM and SH conditions. This enrichment may be attributed to the faster decomposition rate of *T. chinensis* litter under moderate- and high-salinity conditions. These results are consistent with the findings of Song et al. [57], who reported that straw addition significantly increased *Pseudeurotium* abundance and promoted the proliferation of soil fungi associated with straw decomposition in a study conducted at the Shanghai Academy of Agricultural Sciences. Similarly, Razgulin and Voronin [58] reported that *Trichoderma* dominated the fungal community of saprotrophic fungi and promoted litter decomposition.

Elevated soil salinity accelerated the decomposition of *T. chinensis* litter and increased the content of soil available nutrients but caused a sharp decline in microbial alpha diversity and led to a highly convergent community structure. This restructured community was enriched with halotolerant, oligotrophic functional groups, predominantly from the phyla Proteobacteria and Actinobacteriota. We propose that *T. chinensis* litter may provide a suitable carbon source for these microbial groups [59,60]. While increased salinity conditions the ecosystem through the deterioration of soil physical structure and the toxicity of salt ions, a synergistic mechanism emerges under optimal salinity conditions. This synergy involves the enrichment of soil available nutrients and soil enzyme activities driven by *T. chinensis* litter decomposition and the functional specialization of the microbial community (dominated by efficient, salt-tolerant decomposers such as Proteobacteria) [61]. This interplay may exemplify an adaptive feedback mechanism within the saline–alkaline ecosystem. In future studies, we will employ functional approaches, including metagenomics, soil extracellular enzyme assays, microbial respiration measurements, and stable isotope tracing, to validate this mechanism [62,63,64,65].

## 4. Materials and Methods

### 4.1. Design of the Simulated Experimental Pools

We established the simulated experimental site at the experimental base of the Shandong Key Laboratory of Eco-Environmental Science for the Yellow River Delta in China, according to Dong et al.’s [66] standard for classifying saline–alkali land types based on soil salinity. In terms of the total salinity content in agricultural soils, coastal saline–alkali soils are categorized mainly into nonsaline–alkali soils (<0.2%), slightly saline–alkali soils (0.2–0.4%), moderately saline–alkali soils (0.4–0.8%) and highly saline–alkali soils (1.0–1.5%). In September 2022, we conducted a simulated control experiment by irrigating soils with saline water at varying concentrations. Four experimental pools were established with soil salinities of 0.1% (CK, control), 0.4% (SS, slight salinity), 0.8% (SM, moderate salinity), and 1.2% (SH, high salinity). Soil salinity was determined by measuring the soluble salt content. Salinity gradients were established by adding a salt solution with an ionic composition similar to that of the local saline–alkaline soil, containing NaCl, Na_2_SO_4_, and NaHCO_3_, with Na^+^ as the dominant cation and Cl^−^ and SO_4_^2−^ as the major anions. Each treatment was replicated three times, resulting in a total of 12 experimental pools, each measuring 1 m × 1 m × 0.8 m (length × width × depth) with a volume of 0.80 m^3^. To prevent salt loss, the bottoms and edges of all pools were sealed, and a 0.13 m space was left at the top. To ensure statistical reliability, the positions of the experimental pools were arranged using a completely randomized design. The characteristics of the experimental soil are detailed in a previous study [19].

### 4.2. Collection and Treatment of T. chinensis Litter Samples

In March 2023, we collected *T. chinensis* litter used as decomposition material from a forest in Binzhou Harbor, Binzhou City, Shandong Province, located within the Yellow River Delta (118°3′15″–118°4′32″ E, 38°9′51″–38°10′44″ N). Upon collection, the litter samples were conveyed to the laboratory, where they were cleansed of impurities and air-dried until a constant weight was attained. Subsequently, 15 g portions of nondecomposed (NDL), semidecomposed (SDL), and already decomposed (ADL) litter were separately enclosed in nylon mesh bags (dimensions: 25 cm × 15 cm; mesh aperture: 0.5 mm) for the decomposition experiment [19,67].

### 4.3. Experimental Setup and Sample Collection

**Figure 12:**
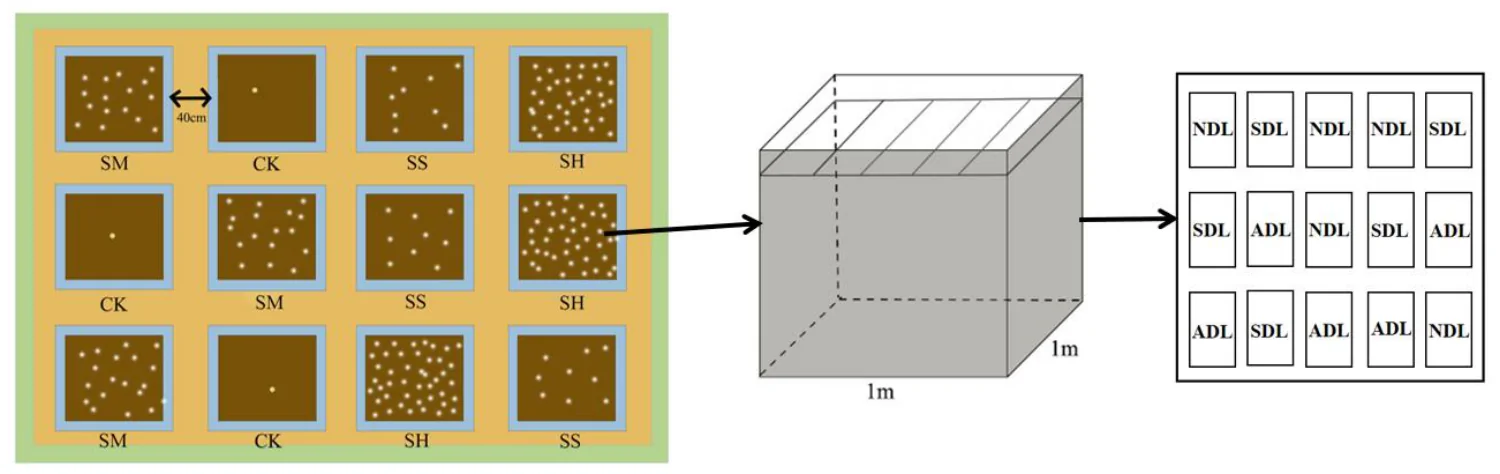
Experimental design layout showing the four salinity treatments (CK, SS, SM, SH) with three replications each. Notes: CK: control; SS: slight salinity; SM: moderate salinity; SH: high salinity; NDL: nondecomposed litter; SDL: semidecomposed litter; ADL: already decomposed litter.

In March 2023, each experimental pool was subdivided into 15 units. Litter bags containing NDL, SDL, and ADL were then randomly assigned to these units, with five replicates per litter type. To prevent cross-contamination between adjacent treatments, PVC partitions (80 cm × 30 cm and 80 cm × 20 cm) were inserted to a depth of 80 cm. Litter samples were randomly distributed across all 12 experimental pools (Figure 12). The full experimental design included four salinity levels, three experimental pools replicates, three litter types, and five sample replicates, totaling 180 decomposition bags. Collections occurred every six months over five sampling events. Bags were arranged parallel without overlap, with the decomposition period commencing on the placement date [19].

After 180 days of decomposition in September 2023, we retrieved litter and soil samples for analysis. For each combination of decomposition degree (three levels) and soil salinity (four levels), one litter bag was collected per replicate (three replicates), totaling 36 bags. Soil samples were taken from the underlying 0–10 cm layer. In the laboratory, litter was cleaned of adhering soil and debris, dried at 65 °C, pulverized, and sieved to 60-mesh. The decomposition rate was derived from the mass loss equation: (initial—residual)/initial × 100%. Soil samples were either preserved at −20 °C for microbial sequencing or air-dried, ground, and sieved to 20 mesh for physicochemical analyses.

### 4.4. Indicator Measurement

Soil physicochemical indicators: The soil bulk density and soil saturated water content were determined using the ring knife method, while the soil salt content was measured gravimetrically. The soil electrical conductivity (EC) was determined via the potentiometric method. A flow injection analyzer was used to quantify the concentrations of NH_4_^+^-N and NO_3_^−^-N. The soil available phosphorus (AP) content was determined via the molybdenum–antimony–scandium colorimetric method, while the soil available potassium (AK) content was determined by flame spectrophotometry.

Soil DNA extraction, amplification, and high-throughput sequencing: Total microbial community genomic DNA was extracted from soil samples according to the manufacturer’s instructions for the E.Z.N.A.^®^ Soil DNA Kit (Omega Bio-tek, Inc., Norcross, GA, USA). The quality of the extracted genomic DNA was assessed by 1% agarose gel electrophoresis, while its concentration and purity were determined using a NanoDrop 2000 ultramicro spectrophotometer (Thermo Fisher Scientific Inc., Wilmington, DE, USA). Using the extracted DNA as a template, specific target regions were amplified via polymerase chain reaction (PCR). The hypervariable V3-V4 region of the bacterial 16S rRNA gene was amplified using the primer pair 338F (5′-ACT CCT ACG GGA GGC AGC AG-3′) and 806R (5′-GGA CTA CHV GGG TWT CTA AT-3′). The fungal internal transcribed spacer (ITS) region was amplified using the primer pair ITS1F (5′- CTT GGT CAT TTA GAG GAA GTAA-3′) and ITS2R (5′- GCT GCG TTC TTC ATC GAT GC-3′). All PCR amplifications were performed on an ABI GeneAmp^®^ 9700 PCR thermal cycler (Applied Biosystems, Foster City, CA, USA). The PCR mixture (20 μL) contained 2× Phanta Max Master Mix. The PCR products were separated on a 2% agarose gel, and target bands were excised and purified using a PCR Clean-Up Kit (Shanghai Majorbio YuHua Bio-pharm Technology Co., Ltd., Shanghai, China) according to the manufacturer’s protocol. The purified products were quantified using a Qubit 4.0 fluorometer (Thermo Fisher Scientific Inc., Waltham, MA, USA). Sequencing libraries were constructed from the purified amplicons using the NEXTFLEX Rapid DNA-Seq Kit (Bioo Scientific Corp., Austin, TX, USA) through the following steps: (1) adapter ligation, (2) removal of self-ligated adapter fragments using magnetic bead-based purification, (3) library template enrichment via limited-cycle PCR amplification, and (4) final library recovery by purifying the PCR products with magnetic beads. Finally, paired-end sequencing (2 × 300 bp) was performed on an Illumina MiSeq PE300 platform (Majorbio Biopharm Technology Co., Ltd., Shanghai, China).

### 4.5. Data Processing

Differences in litter decomposition rates, soil physicochemical properties, and microbial alpha diversity indices across salinity treatments were assessed using two-way analysis of variance (ANOVA), one-way ANOVA, and the Duncan method (*p* < 0.05). Mantel tests were conducted to examine relationships between the soil salinity condition matrix and various environmental factor matrices. Correlations among environmental factors were evaluated using Spearman correlation analysis. To explore direct and indirect pathways among variables, structural equation modeling (SEM) was constructed and analyzed with the lavaan and semPlot packages in R (version 4.3.2). We used Microsoft Excel 2021 to organize the data and SPSS 27.0 for statistical analysis. Visualizations were prepared using the ggplot2 package in R Studio 2025, along with Origin 2018 and Microsoft PowerPoint 2021.

The specific code of the SEM method is as follows:

library(lavaan)
model <- ‘
# measurement model (latent and observed variables)
Latent1 = ~ item1 + item2 + item3
Latent2 = ~ item4 + item5 + item6
# structural model (relationship between latent variables)
Latent1 ~ latent2

## 5. Conclusions

As soil salinity increased, the decomposition rate of *T. chinensis* litter and soil bulk density tended to increase, whereas the soil saturated water content tended to decrease. The soil salt content and EC followed the order of SM > SH > SS > CK. Overall, the content of soil available nutrients increased. Soil microbial alpha diversity and the number of ASVs decreased significantly, and the beta diversities gradually overlapped. The relative abundance of dominant bacterial and fungal phyla, along with the number of dominant genera (e.g., those of Proteobacteria, *Ascomycota*, *Marinobacter*, and *Madurella*) increased.

SEM revealed that soil salinity had a positive effect on the decomposition rate of *T. chinensis* litter but a negative effect on soil physical properties, which subsequently influenced soil available nutrient contents and ultimately significantly affected bacterial diversity. In contrast, the degree of litter decomposition did not significantly affect the litter decomposition rate, soil physicochemical properties, or microbial community structure. In summary, soil salinity not only serves as a key driver of litter decomposition but also influences soil nutrient cycling processes by altering the soil microenvironment. The decomposition of *T. chinensis* litter notably increased soil available nutrients and strongly increased the selective filtering capacity of microbial communities in moderately to highly saline‒alkaline coastal soils.

These findings identify soil salinity as a dominant environmental filter shaping litter decomposition, soil available nutrients, and microbial community assembly in saline–alkaline coastal ecosystems, and suggest that litter-derived nutrient inputs may reinforce salinity-driven microbial selection, with important implications for nutrient turnover in these systems. Nevertheless, because taxonomic shifts do not necessarily reflect functional changes, this mechanism requires further verification. These results highlight the importance of considering salinity gradients and plant–microbe interactions in the restoration of coastal saline–alkaline ecosystems, and future studies should use functional approaches such as metagenomics, soil extracellular enzyme assays, microbial respiration measurements, and stable isotope tracing to validate the underlying mechanisms. In addition, continuous hydrological monitoring and mass-balance approaches should be integrated to quantify nutrient and salt budgets in this open system.

## Data Availability

The original contributions presented in the study are included in the article. Further inquiries can be directed to the corresponding author.
